# Higher serum PFDA is associated with increased non-melanoma skin cancer odds in NHANES 2003–2018

**DOI:** 10.17305/bb.2026.13621

**Published:** 2026-04-03

**Authors:** Sundus Siddiqui, Hiba Siddiqui, Karim Nagi, Mohammed Imad Malki

**Affiliations:** 1 College of Medicine, QU Health, Qatar University, Doha, Qatar

**Keywords:** National Health and Nutrition Examination Survey, NHANES, per- and polyfluoroalkyl substances, PFAS, skin cancer

## Abstract

Per- and polyfluoroalkyl substances (PFAS) are environmentally persistent chemicals commonly employed in heat- and water-resistant applications. Experimental evidence indicates a biological plausibility for PFAS-related carcinogenic effects through mechanisms such as oxidative stress and immunomodulation; however, epidemiological evidence regarding skin cancers remains limited. This study aimed to investigate the association between serum concentrations of three understudied PFAS—perfluorodecanoic acid (PFDA), perfluoroundecanoic acid (PFUnDA), and 2-(N-methyl-perfluorooctane sulfonamido) acetic acid (2-(N-methyl-PFOSA) acetate)—and the incidence of non-melanoma skin cancer (NMSC) and melanoma among U.S. adults. We conducted a cross-sectional analysis utilizing data from eight NHANES cycles (2003–2018), which included 5,934 adults aged 20 years and older with complete data. Skin cancer outcomes were determined based on self-reported physician diagnoses, while PFAS concentrations were assessed in serum samples. We employed multivariable logistic regression, adjusting for age, sex, race/ethnicity, body mass index, smoking status, and NHANES cycle. PFAS exposure was evaluated using tertiles, detectable/undetectable status, and log-transformed continuous measures. Additionally, exploratory age- and sex-stratified analyses were conducted using Firth penalized logistic regression, and multiple imputation was applied to address potential selection bias due to missing covariates. Compared to the lowest tertile, PFDA in the second tertile was associated with an increased likelihood of NMSC (adjusted odds ratio [aOR] 1.73, 95% uncertainty interval [UI] 1.01–2.89; *P* ═ 0.048), although no clear dose–response trend was observed across tertiles. Among adults aged 60 years and older, PFDA in the second tertile was linked to higher odds of NMSC (aOR 2.29, 95% UI 1.29–4.05; *P ═* 0.004). Associations for PFUnDA and 2-(N-methyl-PFOSA) acetate were generally inconsistent across exposure metrics, and analyses for melanoma did not reveal a definitive association. These findings suggest a potential link between higher PFDA exposure and NMSC, particularly in older adults, underscoring the necessity for ongoing PFAS monitoring and well-designed prospective studies to elucidate the temporal relationship and causality.

## Introduction

Per- and polyfluoroalkyl substances (PFAS) are a class of compounds characterized by a carbon chain bonded to multiple fluorine atoms, commonly referred to as a perfluorinated or polyfluorinated backbone. Variations in their chemical properties stem from the different functional groups attached to the termini of these chains [1]. Despite their widespread use and environmental persistence, a universally accepted definition of PFAS remains elusive. The Organization for Economic Co-operation and Development (OECD) defines PFAS as fluorinated compounds containing at least one fully fluorinated methyl (–CF_3_) or methylene (–CF_2_–) carbon atom, devoid of hydrogen, chlorine, bromine, or iodine bonds. This definition encompasses a broad and diverse group of chemicals characterized by perfluorinated carbon structures [2–4]. The Interstate Technology and Regulatory Council (ITRC) offers an alternative definition, describing PFAS as compounds formed by carbon atoms linked together and bonded to fluorine atoms at most or all possible bonding positions. This definition emphasizes the exceptional strength of carbon–fluorine bonds, a critical feature that accounts for the persistence of PFAS and their extensive applications [5].

PFAS represent a large and diverse class of synthetic chemicals first introduced in the 1940s. Since then, they have been extensively utilized in consumer products and industrial applications due to their resistance to heat, water, and oil [6]. PFAS are integral to water- and stain-resistant textiles, carpets, household cleaners, paints, and fire-fighting foams. Certain PFAS are also employed in non-stick cookware, food packaging materials, and food processing equipment [7]. Additionally, increasing numbers of contaminated sites are being identified globally, particularly in the United States (U.S.) [8, 9]. Human exposure to PFAS primarily occurs via drinking water, indoor dust, air, food, and soil [10, 11]. Between 2013 and 2015, PFAS levels exceeding reporting thresholds were detected in 194 out of 4,864 tested public water systems in the U.S. Approximately 6 million people received drinking water from 66 of these systems, resulting in detectable serum PFAS concentrations [12].

Consequently, concerns regarding the health impacts of PFAS are escalating, prompting studies to explore their effects on various aspects of human health. Literature indicates associations between PFAS exposure and several adverse health outcomes, including altered immune function, thyroid dysfunction, liver pathologies, insulin resistance, dyslipidemia, renal dysfunction, and reproductive complications [13]. Proposed biological mechanisms through which PFAS may influence these adverse outcomes include the activation of peroxisome proliferator-activated receptors (PPARs), disruption of lipid and bile acid metabolism, interference with endocrine signaling, immunomodulation, and oxidative stress [14]. Experimental studies suggest that PFAS induce oxidative stress and generate reactive oxygen species (ROS), which may contribute to DNA damage and impaired DNA repair in skin cells, indicating potential biological plausibility for an association between PFAS exposure and skin cancer [15]. Furthermore, PFAS demonstrate immunomodulatory effects, potentially compromising cutaneous immune surveillance and the elimination of transformed cells [16]. The endocrine-disrupting effects of PFAS may further impact skin cell growth and immune responses. Long-chain PFAS, such as perfluorodecanoic acid (PFDA) and perfluoroundecanoic acid (PFUnDA), exhibit high persistence and bioaccumulation, while precursor compounds such as 2-(N-methyl-perfluorooctane sulfonamido) acetic acid (2-(N-methyl-PFOSA) acetate) can be metabolized to perfluorooctane sulfonate (PFOS), extending internal exposure duration [17].

Despite the increasing detection of these compounds in human populations, epidemiologic data regarding their health effects remain limited, underscoring the need for further research, particularly concerning carcinogenic effects and skin cancers [6, 18]. Skin cancers are broadly categorized into two subtypes: melanoma and non-melanoma skin cancer (NMSC), collectively accounting for approximately half of all malignancies [19]. In the U.S., over one million NMSC cases and more than 40,000 melanoma cases are diagnosed annually. Although melanoma constitutes only about 4% of all skin cancer cases, it accounts for the majority of skin cancer-related fatalities [19]. The incidence of NMSC is steadily increasing [20, 21]. NMSC encompasses basal cell carcinoma (BCC) and squamous cell carcinoma (SCC) [22]. While melanoma and NMSC are influenced by various factors, prolonged and unprotected exposure to natural ultraviolet (UV) radiation or artificial UV sources is widely recognized as the primary cause of skin cancer [22]. NMSCs typically develop in sun-exposed areas such as the head, neck, hands, and trunk [23]. The risk is particularly heightened in individuals with fair skin and those prone to sunburn [24]. Additional risk factors include prior radiation exposure, a weakened immune system, and exposure to certain chemicals, such as arsenic [25].

The U.S. National Health and Nutrition Examination Survey (NHANES) datasets serve as a valuable resource for investigating potential associations between exposure to various PFAS compounds and skin cancer, despite the cross-sectional nature of the data. This study primarily aimed to examine the potential association between NMSC and exposure to three specific PFAS compounds not previously assessed: PFDA, PFUnDA, and 2-(N-methyl-PFOSA) acetate. The International Agency for Research on Cancer (IARC) has evaluated the carcinogenicity of select PFAS; however, the specific compounds studied here have not yet been individually classified by IARC. Currently, IARC classifies perfluorooctanoic acid (PFOA) as carcinogenic to humans (Group 1) and PFOS as possibly carcinogenic to humans (Group 2B), raising broader concerns about PFAS-related health effects [26]. This study also explored the association between these chemicals and melanoma skin cancer. Additionally, we aimed to investigate differences in the association between PFDA and NMSC based on age and sex through subgroup analyses and formal interaction tests. These findings may inform future prospective epidemiological studies on PFAS exposure and its potential role in skin cancer development.

## Materials and methods

### Study design and sample

This cross-sectional study utilized publicly available data from NHANES. The NHANES dataset is compiled from non-hospitalized participants across 15 counties per NHANES cycle from each of the 50 U.S. states, managed by the National Center for Health Statistics (NCHS). Demographic information, imaging and clinical tests, physical examinations, laboratory investigations, and questionnaires are collected biennially. The NHANES study protocol has received approval from the Institutional Review Board (IRB) of the NCHS to ensure ethical oversight, and written informed consent was obtained from all participants.

This study analyzed data from eight NHANES cycles conducted between 2003 and 2018, encompassing a total of 44,790 adult participants aged 20 and older. Pregnant individuals (254 participants) were excluded based on questionnaire data and urinary pregnancy test results. Additionally, individuals with incomplete data on other covariates were omitted. Consequently, the final analytical sample comprised 5,934 participants for NMSC, as illustrated in Figure 1, and 5,873 participants for melanoma skin cancer, as detailed in Table S1.

**Figure 1.**
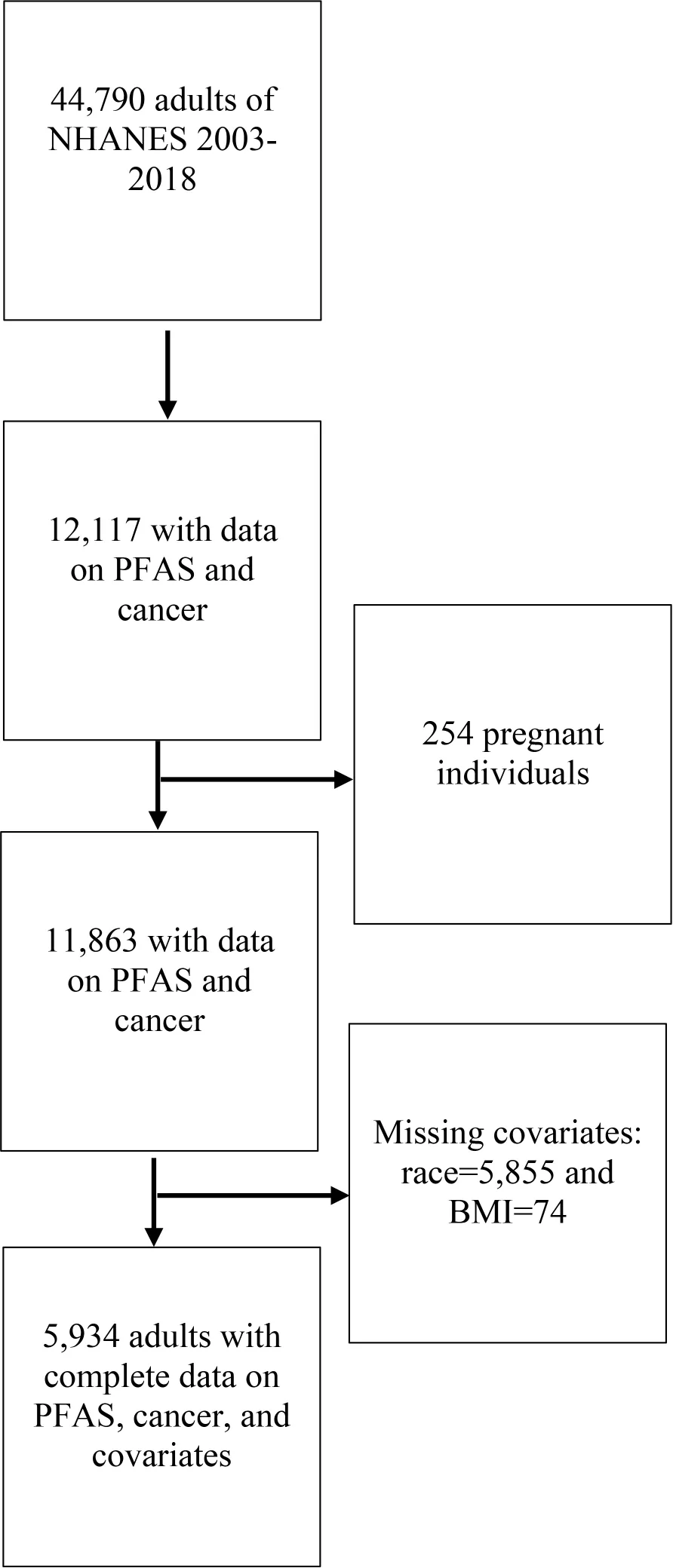
**Flowchart illustrating the NHANES participants who fulfilled the inclusion criteria.** **Abbreviations:** BMI, body mass index; NHANES, National Health and Nutrition Examination Survey; PFAS, per- and polyfluoroalkyl substances.

### Exposure measure: Serum per- and polyfluoroalkyl levels

Eligible serum samples from participants aged 12 and older were analyzed by NHANES for various PFAS using online solid phase extraction coupled with high-performance liquid chromatography-turboionspray ionization-tandem mass spectrometry. This method allows for rapid detection of PFAS in human serum, with limits of detection in the low parts-per-billion (ppb or ng/mL) range. It is important to note that while PFAS concentrations were available for participants aged ≥12 years, analyses were restricted to participants aged ≥20 years in this study, consistent with the age range for skin cancer assessment.

Detection limits remained consistent across all analytes in the dataset. If an analyte’s measurement fell below the lower limit of detection, an imputed fill value was assigned in the results field, calculated as the lower limit of detection (LLOD) divided by the square root of 2 (LLOD/√2). The LLOD for each of the three PFAS compounds in this study was set at 0.10. The NHANES dataset utilized this LLOD as the cutoff to classify participants as having detectable or undetectable serum PFAS levels. We employed this binary variable to conduct six independent association analyses, examining the relationship between each PFAS and the two types of skin cancer.

To standardize quality control, the NCHS developed and disseminated a protocol for both Centers for Disease Control and Prevention (CDC) and contract laboratories. This protocol incorporates Westgard rules [27] for evaluating NHANES specimens. Laboratories are mandated to submit quarterly progress reports detailing any issues related to specimen shipment or receipt, control pool summary statistics, quality control charts, equipment calibration, reagents utilized, and any special circumstances. The NCHS reviews these reports to identify data trends or anomalies, and laboratories must address any identified concerns.

All manufacturer-recommended quality control procedures were strictly adhered to. Results from all assays met the Division of Laboratory Sciences’ standards for accuracy and precision, aligning with quality control benchmarks such as those outlined by the Westgard rules [28].

### Outcome measure: Skin cancers

This study examined skin cancers, specifically NMSC and melanoma. The classification of these conditions was derived from responses obtained through the NHANES questionnaires for each survey cycle. Cancer status was determined based on participants’ responses to a question regarding whether they had ever been informed of a cancer diagnosis. If a participant confirmed a cancer diagnosis, a follow-up question was posed to ascertain the type of cancer. This data facilitated the classification of participants as having either NMSC or melanoma. For participants with multiple cancer types, NHANES reported each type as a separate variable, and responses to these variables were combined to classify the type of skin cancer.

### Covariates

Self-reported demographic variables, including age (in years), sex, race (categorized as White, Hispanic, and other non-Hispanic), and NHANES cycle, were included as covariates in the outcome analysis. Body mass index (BMI) was derived from examination data on body measurements. Smoke exposure was classified using both self-reported questionnaire responses and laboratory data. Participants were identified as active smokers if they indicated current smoking, reported having smoked more than 100 cigarettes in their lifetime, or exhibited serum cotinine levels of 10 ng/mL or higher. Three additional covariates were utilized as prognostic variables: diabetes, sunburn, and urinary arsenic acid levels [29]. Diabetes was classified based on questionnaire data regarding diabetes, drug treatment for diabetes, and laboratory data indicating elevated glycohemoglobin (≥6.5%). Self-reported data on sunburn status included whether a person experienced sunburn in the past year, the number of times they were sunburned, and skin reactions to sun exposure after a period of non-exposure (ranging from mild to severe burning). Urinary arsenic acid levels were also incorporated, with a natural logarithm transformation applied to urinary creatinine values to adjust for urinary dilution.

**Table 1:** Demographic and health characteristics of the adult sample across eight NHANES cycles (2003-2018) categorized by non-melanoma skin cancer status, *n* ═ 5,934

**Characteristic**	**Category**	**No non-melanoma skin cancer**	**Non-melanoma skin cancer**	***P* value**
Total *n* (%)		5,830 (98.2)	104 (1.8)	
Mean age (SD)		48.1 (17.2)	70.2 (11.7)	<0.001
Age category, *n* (%)	20-59 (Adults)	4,102 (70.4)	22 (21.2)	<0.001
	≥60 (Senior)	1,728 (29.6)	82 (78.8)	
Sex, *n* (%)	Males	2,840 (48.7)	61 (58.7)	0.044
	Females	2,990 (51.3)	43 (41.3)	
Ethnicity, *n* (%)	White	1,994 (34.2)	97 (93.3)	<0.001
	Hispanic	1,455 (25.0)	5 (4.8)	
	Other	2,381 (40.8)	2 (1.9)	
Smoking, *n* (%)	No	3,269 (56.1)	47 (45.2)	0.027
	Yes	2,561 (43.9)	57 (54.8)	
Diabetes, *n* (%)	No	4,377 (75.1)	71 (68.3)	0.112
	Yes	1,453 (24.9)	33 (31.7)	
Body mass index category, *n* (%)	Underweight, <18.5	97 (1.7)	2 (1.9)	0.863
	Normal, 18.5 to <25	1,585 (27.2)	32 (30.8)	
	Overweight, 25 to <30	1,859 (31.9)	31 (29.8)	
	Obese, ≥30	2,289 (39.3)	39 (37.5)	
Mean body mass index (SD)		29.4 (7.2)	28.6 (5.9)	0.254
Chronic kidney disease, *n* (%)	No	5,080 (88.1)	87 (86.1)	0.553
	Yes	688 (11.9)	14 (13.9)	
Sunburn, *n* (%)	No	3,884 (66.6)	85 (81.7)	0.001
	Yes	1,946 (33.4)	19 (18.3)	
Urinary Arsenic (SD)		20.3 (54.1)	19.8 (34.5)	0.968
Perfluorodecanoic acid (PFDA) Tertile, *n* (%)	1	3,609 (61.9)	56 (53.8)	0.031
	2	908 (15.6)	26 (25.0)	
	3	1,313 (22.5)	22 (21.2)	
Geometric mean serum PFDA (GSD)		0.20 (2.28)	0.21 (2.12)	0.504
PFDA Detectable, *n* (%)	No	1,078 (18.5)	16 (15.4)	0.418
	Yes	4,752 (81.5)	88 (84.6)	
Perfluoroundecanoic acid (PFUnDA) Tertile, *n* (%)	1	3,402 (58.4)	61 (58.7)	0.203
	2	1,134 (19.5)	26 (25.0)	
	3	1,294 (22.2)	17 (16.3)	
Geometric mean serum PFUnDA (GSD)		0.14 (2.26)	0.13 (1.90)	0.380
PFUnDA Detectable, *n* (%)	No	2,548 (43.7)	41 (39.4)	0.383
	Yes	3,282 (56.3)	63 (60.6)	
2-(N-Methyl-perfluorooctane sulfonamido) acetic acid (2-(N-methyl-PFOSA) acetate) Tertile, *n* (%)	1	3,897 (66.8)	50 (48.1)	<0.001
	2	1,176 (20.2)	31 (29.8)	
	3	757 (13.0)	23 (22.1)	
Mean Serum 2-(N-methyl-PFOSA) acetate (SD)		0.12 (2.22)	0.16 (2.32)	<0.001
2-(N-methyl-PFOSA) acetate Detectable, *n* (%)	No	3,048 (52.3)	37 (35.6)	<0.001
	Yes	2,782 (47.7)	67 (64.4)	

Statistical tests utilized to evaluate group differences included independent samples *t*-tests and Mann–Whitney *U* tests, contingent upon the data distribution. Categorical variables were compared using chi-squared tests or Fisher’s exact tests, as applicable. **Abbreviations:** GSD, geometric standard deviation; NHANES, National Health and Nutrition Examination Survey; PFDA, perfluorodecanoic acid; PFUnDA, perfluoroundecanoic acid; SD, standard deviation.

### Ethical statement

The present study utilized de-identified, publicly available data from NHANES, collected under protocols approved by the NCHS IRB. All participants provided written informed consent prior to data collection. The study adhered to the principles of the Declaration of Helsinki and received ethical approval from the NCHS Research Ethics Review Board. Since the NHANES data are publicly accessible and participant consent has already been obtained, additional ethical approval from the Qatar University IRB was unnecessary.

### Statistical analysis

Descriptive statistics were employed to summarize the data, with a table of characteristics depicting the results for the included participants (Table 1). Continuous variables were reported as means and standard deviations (SDs) for approximately symmetric variables or as geometric means and geometric standard deviations (GSD) for right-skewed variables, such as PFAS concentrations. Categorical variables were presented as frequencies or percentages. Multivariable logistic regression analyses were conducted to independently assess the associations between exposure to each of the three PFAS and NMSC (Tables 2 and 3). These analyses were repeated to evaluate the associations between PFAS and melanoma skin cancer (Tables S1 and S2). In essence, we assessed associations between the three PFAS compounds and each skin cancer (NMSC and melanoma) using three classifications for each PFAS exposure variable:

1. Tertiles were created separately for each PFAS exposure variable, as this variable was not normally distributed (Table S3 and Table S4 show characteristics of excluded and included participants and the tertile cut-off values, respectively).
2. A binary exposure variable was utilized based on detectable vs non-detectable serum levels as reported by NHANES.
3. PFAS concentrations were analyzed as continuous variables after natural log transformation to improve model fit, as PFAS was not normally distributed, and to estimate associations per proportional increase in concentration.

**Table 2:** Associations between serum PFAS levels and NMSC utilizing the minimal adjustment set, with variance adjusted for NHANES clustering and stratification

	**Tertiles**	**aOR**	**95% UI**	***P* value**
**PFDA** *n* ═ 5934				
Level	1	1 (base)		
	2	1.73	1.01, 2.98	0.048
	3	1.17	0.70, 1.96	0.544
Continuous		1.17	0.92, 1.50	0.195
Binary#		1.29	0.74, 2.26	0.361
**PFUnDA** *n*= 5934				
Level	1	1 (base)		
	2	1.32	0.86, 2.05	0.203
	3	1.06	0.58, 1.96	0.857
Continuous		1.08	0.83, 1.42	0.548
Binary#		1.34	0.90, 2.00	0.151
**2-(N-methyl-PFOSA) acetate** *n* ═ 5934				
Level	1	1 (base)		
	2	1.09	0.67, 1.77	0.725
	3	0.86	0.52, 1.43	0.551
Continuous		0.92	0.73, 1.16	0.462
Binary#		0.86	0.52, 1.41	0.546

Models account for clustering at the primary sampling unit (PSU) level and stratification through survey design-based variance estimation. Adjusted covariates include age, sex, race, smoke exposure, NHANES cycle and BMI. # Detectable or Undetectable Serum PFAS (as a binary variable) and Non-melanoma Skin Cancer.PFDA tertiles: Wald F-test Prob > F = 0.000; goodness of link *P ═* 0.577. PFDA continuous: Wald F-test Prob > F = 0.000; goodness of link *P ═* 0.890. PFDA#: Wald F-test Prob > F = 0.000; goodness of link *P ═* 0.892. PFUnDA tertiles: Wald F-test Prob > F = 0.000; goodness of link *P ═* 0.801. PFUnDA continuous: Wald F-test Prob > F = 0.000; goodness of link *P ═* 0.828. PFUnDA#: Wald F-test Prob > F = 0.000; goodness of link *P ═* 0.928. 2-(N-methyl-PFOSA) acetate tertiles: Wald F-test Prob > F = 0.000; goodness of link *P ═* 0.719. 2-(N-methyl-PFOSA) acetate continuous: Wald F-test Prob > F = 0.000; goodness of link *P ═* 0.792. 2-(N-methyl-PFOSA) acetate#: Wald F-test Prob > F = 0.000; goodness of link *P ═* 0.858. **Abbreviations:** 2-(N-methyl-PFOSA) acetate, 2-(N-methyl-perfluorooctane sulfonamido) acetic acid; aOR, adjusted odds ratio; BMI, body mass index; NHANES, National Health and Nutrition Examination Survey; NMSC, non-melanoma skin cancer; PFAS, per- and polyfluoroalkyl substances; PFDA, perfluorodecanoic acid; PFUnDA, perfluoroundecanoic acid; PSU, primary sampling unit; UI, uncertainty interval.

**Table 3:** Associations between serum PFAS levels and NMSC using the minimal adjustment set and prognostic variables (Firth penalized logistic regression)

	**Tertiles**	**aOR**	**95%UI**	***P* value**
**PFDA** *n* ═ 1394				
Level	1	1 (base)		
	2	2.02	0.65, 6.27	0.222
	3	2.21	0.70, 6.98	0.176
Continuous		1.74	0.89, 3.40	0.106
Binary#		9.01	0.54, 151.6	0.127
**PFUnDA** *n* ═ 1394				
Level	1	1 (base)		
	2	1.90	0.62, 5.77	0.259
	3	1.54	0.45, 5.26	0.491
Continuous		1.52	0.77, 2.98	0.226
Binary#		2.25	0.74, 6.85	0.152
**2-(N-methyl-PFOSA) acetate** *n* ═ 1394				
Level	1	1 (base)		
	2	0.19	0.03, 1.13	0.068
	3	0.85	0.31, 2.33	0.749
Continuous		0.99	0.61, 1.62	0.971
Binary#		0.51	0.20, 1.31	0.161

Adjusted covariates include age, sex, race, smoking exposure, NHANES cycle, urine creatinine levels, BMI, diabetes status, history of sunburn, and urinary arsenic concentration. # Detectable vs. undetectable serum PFAS as a binary variable and NMSC. **Abbreviations:** 2-(N-methyl-PFOSA) acetate, 2-(N-methyl-perfluorooctane sulfonamido) acetic acid; aOR, adjusted odds ratio; BMI, body mass index; NHANES, National Health and Nutrition Examination Survey; NMSC, non-melanoma skin cancer; PFAS, per- and polyfluoroalkyl substances; PFDA, perfluorodecanoic acid; PFUnDA, perfluoroundecanoic acid; UI, uncertainty interval.

Linear p-trend tests for tertiles were conducted by modeling log-transformed PFAS concentrations as continuous variables, with none of the PFAS compounds exhibiting missing values (Table S5).

Variables potentially associated with both exposure and outcome were identified through a comprehensive literature review and consultation with a subject expert. These variables were incorporated into a directed acyclic graph (DAG), facilitating the identification of a minimal set of covariates necessary to control for confounding. This approach is regarded as the most effective method for selecting covariates in epidemiological analyses [30]. The final minimal adjustment set for both NMSC and melanoma skin cancers included smoking, sex, age, race, and BMI (Figure S1). The NHANES cycle was also controlled for. Additionally, prognostic variables for skin cancer were adjusted to mitigate potential magnification of risk factors [29]. The prognostic factors for skin cancer included diabetes, sunburn, and urinary arsenic levels (Figure S1). Model fit for each regression model was assessed using the Wald F-test to evaluate the overall significance of predictors, with goodness of fit assessed using the link test in Stata.

The primary analysis employed standard logistic regression adjusted for a minimal sufficient adjustment set identified *a priori*. When additional prognostic variables were included, standard logistic regression exhibited sparse-data bias and perfect prediction due to small cell counts and a rare outcome. Therefore, we employed Firth penalized logistic regression, which applies a bias-reducing penalty to the likelihood and provides finite, stable estimates in the presence of separation or sparse data [31]. This expanded model aimed to assess the robustness of results to additional prognostic adjustments rather than serving as the primary causal model. Firth penalized regression was unnecessary for the primary minimally adjusted model, which did not exhibit separation, but became essential when additional prognostic covariates were included, leading to perfect prediction under standard maximum likelihood estimation [31].

Furthermore, we conducted a sensitivity analysis to address potential selection bias due to missing BMI or race. To do this, we performed multiple imputation (MI) using chained equations (20 imputations) [32]. Race (categorical) and BMI (continuous) were imputed using mlogit and linear regression, respectively. Imputed datasets were analyzed using logistic regression, and estimates were pooled according to Rubin’s rules (Table S6) [33].

It is important to note that weights were intentionally omitted from our analysis. Although NHANES provides sampling weights to support population-level inference, these weights are primarily intended to enhance generalizability by making estimates representative of the U.S. population. In the context of this cross-sectional analysis, the primary objective is to examine associations between exposures and outcomes, expressed as adjusted odds ratios (aOR), rather than to generate nationally representative prevalence estimates. As outlined by Murad et al., when the goal is associative inference, internal validity and control of confounding are more critical than population generalizability [34]. The relevance of generalizability depends on the research question, and evidence may remain valid and applicable even when not designed to represent a broader population [34]. Accordingly, this analysis prioritizes internal validity and direct alignment with the study question, and sampling weights were not applied.

This study incorporates recent recommendations on statistical reporting, which emphasize a shift in terminology from confidence intervals (CIs) to uncertainty intervals (UIs) [35]. *P*-values were employed to assess whether the study findings corresponded with a population model assuming no effect (i.e., the null hypothesis) [35]. Additionally, point estimates were presented alongside 95% UIs, which denote the range of model-based hypotheses that would encompass the observed data within the central 95% of their expected value distributions [35]. All statistical analyses were performed using Stata version 18.5 [36].

## Results

### Study sample and demographics

The total sample comprised 44,790 adults in the U.S., recruited between 2003 and 2018. Among these, 12,117 individuals provided data on serum PFAS and NMSC (Figure 1). After excluding 254 participants due to pregnancy, a total of 11,863 individuals remained. Following the removal of participants with missing covariate data (race *N* ═ 5,855 and BMI *N* ═ 74), the final analytical sample for NMSC consisted of 5,934 participants with complete data on exposure, outcome, and covariates. The final sample size for individuals with data on melanoma and PFAS was 5,873 (Table S1).

Among the 5,934 participants with data on NMSC and PFAS levels, 5,830 individuals did not exhibit NMSC, while 104 were diagnosed with NMSC (Table 1). The prevalence of NMSC was higher in males compared to females (58.7% vs. 41.3%, *P ═* 0.044). Most participants had a BMI of 30 or higher, with 39.3% in the no NMSC group and 37.5% in the NMSC group. The geometric mean serum PFDA level was 0.20 ng/mL (GSD=2.28) in the no NMSC group and 0.21 ng/mL (GSD=2.12) in the NMSC group (*P ═* 0.504). The geometric mean serum PFUnDA levels were 0.14 ng/mL (GSD=2.26) and 0.13 ng/mL (GSD=1.90) with *P ═* 0.380 in the no NMSC and NMSC groups, respectively. The geometric mean serum 2-(N-methyl-PFOSA) acetate levels were 0.12 ng/mL (GSD=2.22) in the no NMSC group and 0.16 ng/mL (GSD=2.32) in the NMSC group (*P* < 0.001).

The primary analytic sample was reduced from 44,790 participants to 5,934 due to missing data on race (*n* ═ 5,855) or BMI (*n* ═ 74). Table S3 compares the characteristics of excluded and included participants. Compared to those excluded, included participants were slightly younger (48.4 vs. 50.6 years, *P* < 0.001) and exhibited minor differences in race distributions (*P* ═ 0.012). However, no gender differences were found between included and excluded participants (*P* ═ 0.382). Mean BMI was identical for both groups. These findings suggest that complete-case analysis may introduce selection bias, particularly regarding race and age. Nevertheless, no differences were observed by gender, and mean BMI was consistent across both groups, indicating that key exposure-related characteristics were comparable.

### Association between serum per- and polyfluoroalkyl substances and NMSC using the minimal adjustment set

Table 2 presents independent adjusted associations utilizing the minimal adjustment set in multivariable logistic regression. Compared to the first tertile, participants with serum PFDA concentrations in the second tertile exhibited a 73% increase in odds of NMSC (95% UI: 1.01, 2.89; *P* ═ 0.048). Participants in the third tertile demonstrated a 17% increase in odds of NMSC (95% UI: 0.70, 1.96; *P* ═ 0.544). For serum PFUnDA concentrations, participants in the second tertile had a 32% increase in odds of NMSC (95% UI: 0.86, 2.05; *P* ═ 0.203), while those in the third tertile exhibited a 6% increase in odds of NMSC (95% UI: 0.58, 1.96; *P* ═ 0.857). For serum 2-(N-methyl-PFOSA) acetate levels, those in the second tertile showed a 9% increase in odds of NMSC (95% UI: 0.67, 1.77; *P* ═ 0.725), while a 14% decrease in odds was observed for those in the third tertile (95% UI: 0.52, 1.43; *P* ═ 0.551) compared to the first tertile. Associations between each of the three PFAS, treated as both continuous (log-transformed) and binary (detectable or undetectable serum PFAS level) variables, and NMSC are also detailed in Table 2.

Table S1 illustrates independent adjusted associations between the three different PFAS and melanoma skin cancer utilizing the minimal adjustment set in multivariable logistic regression.

### Association between serum per- and polyfluoroalkyl substances and NMSC using prognostic variables in addition to the minimal adjustment set (firth penalized logistic regression)

Table 3 outlines independent adjusted associations using the minimal adjustment set along with prognostic variables in the Firth penalized logistic regression model. Notably, NHANES measured urinary arsenic in only one-third of the random environmental subsample of participants. Consequently, analyses incorporating prognostic variables, including urinary arsenic, were limited to this subsample, reducing the analytic sample size from 5,934 to 1,394 participants. This reduction reflects NHANES’ planned subsampling design rather than differential missingness or study attrition. Our results indicate that, compared to the first tertile, participants with serum PFDA concentrations in the second tertile had 2% higher odds of NMSC (95% UI: 0.65, 6.27; *P* ═ 0.222), while those in the third tertile had an aOR of 2.21 (95% UI: 0.70, 6.98; *P* ═ 0.176). For serum PFUnDA concentrations, participants in the second tertile demonstrated a 90% increase in odds of NMSC (95% UI: 0.62, 5.77; *P* ═ 0.259), and those in the third tertile exhibited a 54% increase in odds of NMSC (95% UI: 0.45, 5.26; *P ═* 0.491) compared to the first tertile. In contrast, participants with serum 2-(N-methyl-PFOSA) acetate levels in the second tertile exhibited 91% lower odds of NMSC (95% UI: 0.03, 1.13; *P ═* 0.068), while those in the third tertile showed 15% lower odds of NMSC (95% UI: 0.31, 2.33; *P ═* 0.749). Table 3 further presents independent associations between serum PFAS as both continuous and binary variables and NMSC.

Table S2 displays independent adjusted associations between the three different PFAS and melanoma skin cancer utilizing both the minimal adjustment set and prognostic variables in the Firth penalized logistic regression.

### Association between serum PFDA levels and NMSC: Exploratory subgroup analysis by age (firth penalized logistic regression)

Table 4 illustrates the age-stratified associations between serum PFDA tertiles and NMSC. Among participants aged 60 years and older, those in the second tertile of serum PFDA exhibited 2.29 times the odds of NMSC (aOR=2.29, 95% UI: 1.29, 4.05; *P ═* 0.004) compared to the first tertile. Participants in the third tertile had 1.27 times the odds (95% UI: 0.69, 2.31; *P ═* 0.441) of NMSC relative to the first tertile. Conversely, adults aged 20–59 years displayed lower odds of NMSC in the second tertile (aOR=0.63, 95% UI: 0.16, 2.45; *P ═* 0.507) but borderline increased odds in the third tertile (aOR=1.08, 95% UI: 0.36, 3.17; *P ═* 0.896). *P*-values for interaction were derived from logistic regression models incorporating product terms between PFDA levels and subgroup variables, revealing *P ═* 0.260 for PFDA tertiles and *P ═* 0.382 for log-transformed continuous PFDA.

**Table 4:** Exploratory subgroup analysis by age for the association between PFDA and NMSC (Firth penalized logistic regression)

**Age category**	**PFDA**	**aOR**	**95%UI**	***P* value**
**20-59 years** *n*= 4124	Tertile 1	1 (base)		
	Tertile 2	0.63	0.16, 2.45	0.507
	Tertile 3	1.08	0.36, 3.17	0.896
	Continuous	0.95	0.51, 1.78	0.885
**≥60 years** *n*= 1810	Tertile 1	1 (base)		
	Tertile 2	2.29	1.29, 4.05	0.004
	Tertile 3	1.27	0.69, 2.31	0.441
	Continuous	1.25	0.92, 1.69	0.151

Adjusted covariates include age, sex, race, smoke exposure, NHANES cycle, and BMI. The significance level was established at a *P*-value of less than 0.05. The overall *P* value for the interaction between PFDA tertiles and age category is 0.260, as determined by the Wald test for interaction. The overall *P* value for the interaction between log-transformed continuous PFDA and age category is 0.382, also based on the Wald test for interaction. **Abbreviations:** AOR, adjusted odds ratio; BMI, body mass index; NHANES, National Health and Nutrition Examination Survey; NMSC, non-melanoma skin cancer; PFDA, perfluorodecanoic acid; UI, uncertainty interval.

### Association between serum PFDA levels and NMSC: Exploratory subgroup analysis by sex (firth penalized logistic regression)

Table 5 presents sex-stratified associations between serum PFDA tertiles and NMSC. Male participants with PFDA serum levels in the second and third tertiles exhibited odds of NMSC that were 1.53 (95% UI: 0.77, 3.06; *P ═* 0.223) and 1.19 (95% UI: 0.60, 2.37; *P ═* 0.619) times higher, respectively, compared to the first tertile. Among female participants, those in the second and third tertiles demonstrated odds of NMSC that were 2.07 (95% UI: 0.98, 4.37; *P ═* 0.057) and 1.18 (95% UI: 0.52, 2.69; *P ═* 0.695) times higher, respectively, compared to the first tertile. Interaction for PFDA tertiles yielded *P ═* 0.726, and for log-transformed continuous PFDA, *P ═* 0.294. Figure 2 illustrates the results of subgroup analyses by both age and sex.

**Table 5:** Exploratory subgroup analysis by sex for the association between PFDA and NMSC (Firth penalized logistic regression)

**Sex category**	**PFDA**	**aOR**	**95%UI**	***P* value**
**Male** (n ═ 2,901)	Tertile 1	1 (base)		
	Tertile 2	1.53	0.77, 3.06	0.223
	Tertile 3	1.19	0.60, 2.37	0.619
	Continuous	1.00	0.69,1.44	0.997
**Female** (n ═ 3,033)	Tertile 1	1 (base)		
	Tertile 2	2.07	0.98, 4.37	0.057
	Tertile 3	1.18	0.52, 2.69	0.695
	Continuous	1.44	0.96, 2.15	0.079

Adjusted covariates included age, race, smoking exposure, NHANES cycle, and BMI. The significance level was established at a *P* value of <0.05. The overall *P* value for the interaction between PFDA tertiles and sex was 0.726, as determined by the Wald test for interaction. The overall *P* value for the interaction between log-transformed continuous PFDA and sex was 0.294, also based on the Wald test for interaction. **Abbreviations:** AOR, adjusted odds ratio; BMI, body mass index; NHANES, National Health and Nutrition Examination Survey; NMSC, non-melanoma skin cancer; PFDA, perfluorodecanoic acid; UI, uncertainty interval.

**Figure 2.**
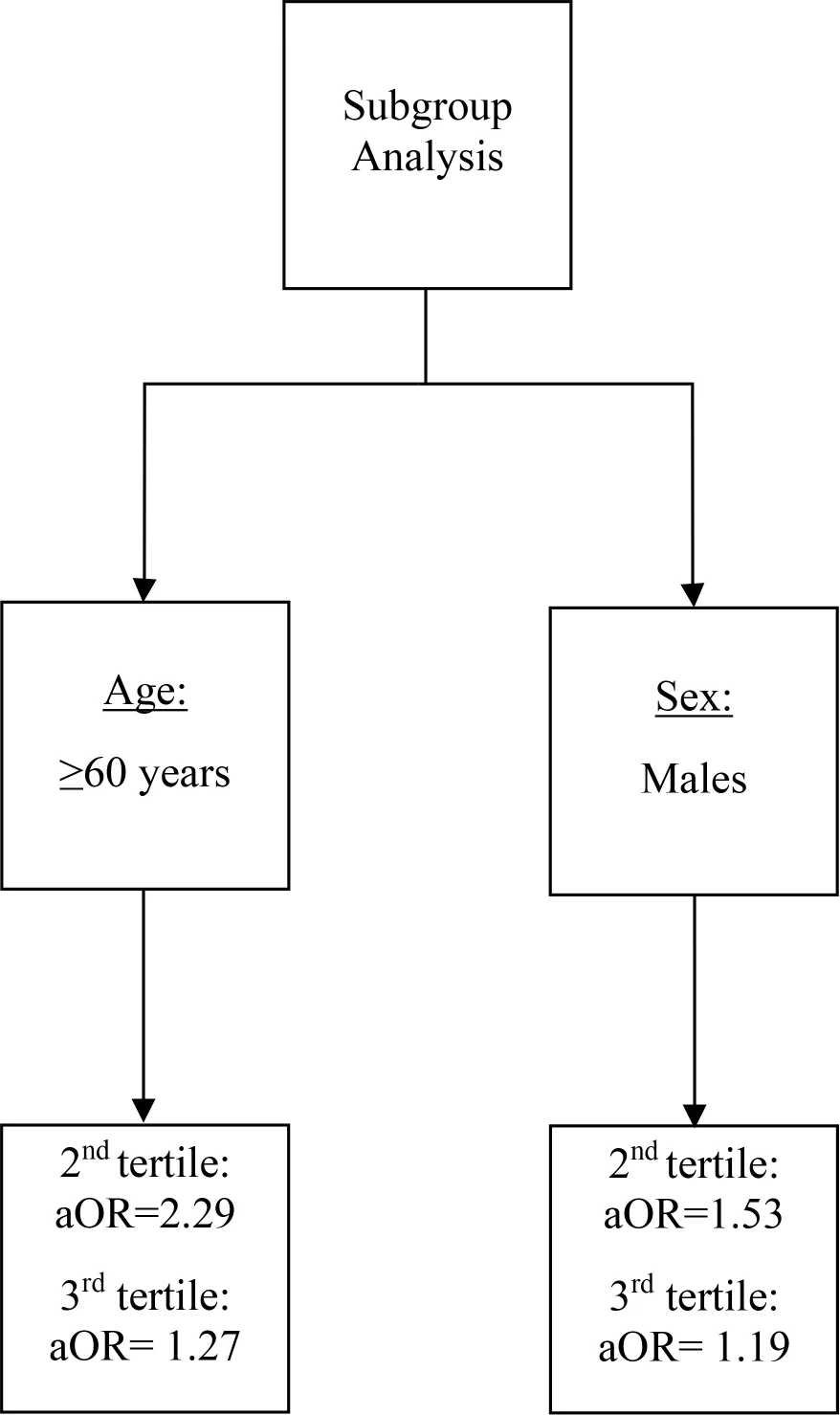
**Schematic representation of subgroup analyses examining the association between serum PFDA levels and NMSC.** **Abbreviations:** aOR, adjusted odds ratio; NMSC, non-melanoma skin cancer; PFDA, perfluorodecanoic acid.

### P-trend tests for tertiles of PFAS

Table S5 indicates that in complete-case analyses involving 5,934 participants, the trends in NMSC odds across tertiles of PFDA, PFUnDA, and 2-(N-methyl-PFOSA) acetate were p-trend = 0.327, 0.625, and 0.666, respectively. Odds ratios per increasing tertile were 1.13 (95% UI: 0.88,1.46) for PFDA, 1.07 (95% UI: 0.81,1.41) for PFUnDA, and 0.94 (95% UI: 0.73,1.23) for 2-(N-methyl-PFOSA) acetate, indicating no clear dose-response relationship.

### Sensitivity analysis: Multiple imputation for the association between serum per- and polyfluoroalkyl substances and NMSC using the minimal adjustment set

Table S6 presents the results of multiple imputation. In the complete-case analysis (*N* ═ 5,934), participants in the second tertile of PFDA exhibited increased odds of NMSC compared to the lowest tertile (aOR 1.73, 95% UI 1.01, 2.98; *P ═* 0.048). Following multiple imputation to include participants with missing BMI or race data (*N* ═ 11,863), this association was attenuated (aOR 1.34, 95% UI 0.92, 1.93; *P ═* 0.123), while the odds for the third tertile remained consistent (aOR 1.15, 95% UI 0.83, 1.59). This discrepancy may reflect an overestimation in complete-case analyses due to differential exclusion related to missing BMI or race data for PFDA in the second tertile. Nonetheless, results from multiple imputation analyses for other exposures and models were generally consistent with those from complete-case analyses. For instance, the aORs for continuous PFDA exposure were 1.15 (95% UI 0.96, 1.37; *P ═* 0.128) using multiple imputation compared to 1.17 (95% UI 0.92, 1.50; *P ═* 0.195) in complete cases; for binary exposure, the aORs were 1.22 (95% UI 0.84, 1.76; *P ═* 0.301) vs 1.29 (95% UI 0.74, 2.26; *P ═* 0.361). For PFUnDA, multiple imputation analyses yielded aORs of 1.06 (95% UI 0.71, 1.59) and 1.23 (95% UI 0.91, 1.67) for tertile 3 and binary exposures, respectively, which were similar to complete-case estimates. For 2-(N-methyl-PFOSA) acetate, aORs were consistent across both analyses: continuous exposure aOR 0.99 (95% UI 0.85, 1.16) vs 0.92 (95% UI 0.73, 1.16). These findings suggest that selection bias due to missing BMI and race is unlikely to have materially influenced our results.

## Discussion

Utilizing NHANES data from 2003–2018, we found that higher PFDA levels were associated with increased odds of NMSC. This association persisted after adjusting for variables such as age, sex, race, smoking exposure, NHANES cycle, and BMI. However, analyses of PFUnDA and 2-(N-methyl-PFOSA) acetate yielded inconclusive results regarding their potential associations with NMSC.

An expanding body of epidemiological evidence has assessed the potential links between PFAS exposure and various cancer outcomes. Numerous studies have explored the associations between PFAS and several cancer types, including bladder, thyroid, esophageal malignancies [37, 38], colorectal [39], and melanoma [40]. However, the evidence concerning the role of PFAS in the development of NMSC remains limited yet increasingly relevant. A recent dose-response meta-analysis encompassing 40 studies with 748,188 participants found that overall PFAS exposure is linked to an elevated risk of genitourinary cancers, with PFDA showing a dose-dependent increase in both overall and breast cancer risk. Additional associations were identified for PFOS and elevated PFOA levels in breast cancer patients [41]. Another comprehensive review of PFAS epidemiology reported testicular and kidney cancers as the sites with the most consistent evidence of association with PFAS exposure, while evidence for prostate cancer was less robust or inconsistent across studies [42]. Importantly, much of the existing literature has primarily focused on cancer types other than NMSC. In this context, our investigation of PFUnDA and 2-(N-methyl-PFOSA) acetate concerning NMSC represents a novel contribution by extending PFAS cancer research to lesser-studied congeners and to NMSC specifically. This focus addresses a gap in the evidence base and suggests avenues for further research on diverse PFAS congeners and a broader array of cancer outcomes.

Our results contrast with findings from a recent study analyzing data from the Surveillance, Epidemiology, and End Results (SEER) Program. While our analysis indicated an increase in aOR, particularly in the second tertile of PFDA (aOR = 1.73; 95% UI: 1.01, 2.98; *P ═* 0.048), the published study reported a decrease in skin cancer risk, with an incidence rate ratio (IRR) of 0.89 (95% UI: 0.84, 0.93; *P* < 0.01). This discrepancy may stem from differing methodologies: their adjusted variables included county-level socioeconomic status (SES) indicators, urbanicity, smoking rates, obesity, and air pollution, without evidence of utilizing a DAG. Moreover, they employed negative binomial regression for their analysis [37, 38], whereas our study utilized multivariable logistic regression and Firth’s penalized logistic regression models.

Our findings align with a recent NHANES-based study covering the years 2003–2018, which examined the association between four PFAS compounds—excluding the three analyzed in our study—and NMSC among other malignancies. Similar to our results, that paper concluded that NMSC was associated with only one PFAS compound, PFOA. They conducted multivariable logistic regression with the time of NMSC diagnosis restricted to ten years prior to the survey, yielding an aOR of 1.24 (95% UI: 0.99,1.56) for each one-unit increase in the natural logarithm of serum PFOA. Conversely, their findings regarding PFOS, perfluorohexanesulfonic acid (PFHxS), and perfluorononanoic acid (PFNA) were inconclusive, with odds ratios and UIs of 1.08 (95% UI: 0.92,1.28), 1.14 (95% UI: 0.94–1.40), and 1.06 (95% UI: 0.85–1.31) respectively [18]. Thus, these findings suggest consistent evidence that certain PFAS compounds may be implicated in the pathogenesis of NMSC.

The plausibility of our findings is supported by animal studies investigating the effects of PFAS on immune function. A recent review suggests that PFAS, particularly PFOS and PFOA, reduce both circulating and resident white blood cell (WBC) counts in the spleen and thymus of animal models [16]. This aligns with previous research indicating that neutrophil, monocyte, and lymphocyte counts were lower in patients with any form of skin cancer, including BCC and SCC [43]. That study also noted the lowest neutrophil-to-lymphocyte ratio among patients with BCC [43]. These results imply that diminished immune defense, potentially linked to reduced WBC counts associated with PFAS exposure, could contribute to the development of various skin cancers.

Another study elucidated the connection between PFAS exposure and cytokine activity modulation. This study revealed that several Th1 cytokines, including tumor necrosis factor alpha (TNF-α) and IL-8, were downregulated due to PFAS exposure. Notably, TNF-α exhibited a significant inverse association with serum levels of PFOA, a trend also observed for PFOS, with IL-8 demonstrating a negative association [44]. These findings are consistent with existing literature, as it is well-documented that the regression of BCC is modulated by tumor suppressor cytokines secreted by the Th1 subset of T-helper cells [45, 46]. Therefore, cytokine dysregulation resulting from PFAS exposure likely leads to decreased cell-mediated immunity, undermining immune surveillance, which is crucial for the elimination of cancerous cells [47].

PFAS-induced epigenetic modifications present another plausible mechanism explaining the association between PFAS exposure and NMSC. A cohort study reported a link between PFAS exposure and increased DNA methylation at 10,598 cytosine-phosphate-guanine (CpG) sites, particularly in male participants, with affected sites associated with genes involved in cancer-related pathways [48]. In particular, hypermethylation of tumor suppressor genes involved in cell signaling pathways that regulate cell proliferation and differentiation has been implicated in carcinogenesis by promoting aberrant cell survival [49]. Our study indicated higher odds of NMSC in males exhibiting serum PFDA levels in the second and third tertiles compared to the first tertile, although the confidence intervals included the null value (aOR = 1.53; 95% UI: 0.77, 3.06; *P ═* 0.223 and aOR = 1.19; 95% UI: 0.60, 2.37; *P ═* 0.619). Furthermore, a recent review suggested that oxidative stress induced by early-life PFAS exposure may serve as a critical mediator linking various epigenetic alterations to adverse health outcomes later in life [50]. Thus, PFAS exposure can lead to detrimental epigenetic modifications that promote cancer development, particularly in males.

This study’s limitations warrant acknowledgment. First, due to its cross-sectional design, establishing a causal link between PFAS and NMSC is not feasible. Second, reliance on self-reported cancer diagnoses introduces potential bias from recall errors or misclassification. Although imputation analyses indicated that our results are unlikely to have been significantly influenced by selection bias, the possibility cannot be entirely discounted. While Firth’s penalized logistic regression was employed in subgroup analyses to mitigate small-sample bias and address issues of sparse data, the limited number of cases may still restrict statistical power and increase uncertainty in effect estimates. Consequently, findings from subgroup analyses should be interpreted cautiously. Finally, although models controlled for key covariates, the potential for residual confounding remains, particularly concerning other PFAS compounds, such as PFOA and PFOS, which were not incorporated into the analytical models. Future studies with larger sample sizes and more comprehensive assessments of PFAS exposure are necessary to confirm and extend these findings.

Our study lays the groundwork for future research aimed at uncovering the biological processes linking PFAS exposure to NMSC through mechanistic and molecular studies. Additionally, large-scale prospective studies are essential to explore temporal relationships. Future investigations should consider a wider range of PFAS compounds and account for regional differences. By leveraging the NHANES dataset, this study provides potentially impactful evidence to inform future risk assessments and public health policy. The observed associations between PFAS and NMSC carry significant implications, extending beyond individual health to influence environmental regulations, agricultural practices, and industrial processes.

## Conclusion

This cross-sectional study, utilizing data from eight cycles of NHANES, identified associations between serum PFAS concentrations and NMSC among participants in the United States. Notably, participants with serum PFDA levels in the second tertile exhibited higher odds of NMSC compared to those in the first tertile. Our findings underscore the necessity for ongoing monitoring and the implementation of control strategies in settings at risk for elevated PFAS exposures.

## Supplemental data

Supplemental data are available at the following link: https://www.bjbms.org/ojs/index.php/bjbms/article/view/13621/4160.

## Data Availability

The data that support the findings of this study are available on request from the corresponding author.

## References

[1] Buck RC (2011). Perfluoroalkyl and polyfluoroalkyl substances in the environment: terminology, classification, and origins. Integr Environ Assess Manag.

[2] Organisation for Economic Co-operation and Development (OECD).

[3] Organisation for Economic Co-operation and Development (OECD). (2021). Reconciling terminology of the universe of per- and polyfluoroalkyl substances: recommendations and practical guidance. Series on Risk Management.

[4] Wang Z (2021). A new OECD definition for per- and polyfluoroalkyl substances. Environ Sci Technol.

[5] Mueller B, et al.

[6] Carlson LM (2022). Systematic evidence map for over one hundred and fifty per- and polyfluoroalkyl substances (PFAS). Environ Health Perspect.

[7] U.S. (2025). Food and Drug Administration. Per- and polyfluoroalkyl substances (PFAS)..

[8] Environmental Working Group.

[9] Steenland K, Winquist A (2021). PFAS and cancer: a scoping review of the epidemiologic evidence. Environ Res.

[10] Sunderland EM (2019). A review of the pathways of human exposure to poly- and perfluoroalkyl substances (PFASs) and present understanding of health effects. J Expo Sci Environ Epidemiol.

[11] U.S. (2024). Environmental Protection Agency. Our current understanding of the human health and environmental risks of PFAS..

[12] Hu XC (2016). Detection of poly- and perfluoroalkyl substances (PFASs) in US drinking water linked to industrial sites, military fire training areas, and wastewater treatment plants. Environ Sci Technol Lett.

[13] Fenton SE (2021). Per- and polyfluoroalkyl substance toxicity and human health review: current state of knowledge and strategies for informing future research. Environ Toxicol Chem.

[14] Hari A (2024). Exposure to PFAS chemicals induces sex-dependent alterations in key rate-limiting steps of lipid metabolism in liver steatosis. Front Toxicol.

[15] Bonato M (2020). PFAS environmental pollution and antioxidant responses: an overview of the impact on human field. Int J Environ Res Public Health.

[16] Ehrlich V (2023). Consideration of pathways for immunotoxicity of per- and polyfluoroalkyl substances (PFAS). Environ Health.

[17] Temkin AM (2020). Application of the key characteristics of carcinogens to per- and polyfluoroalkyl substances. Int J Environ Res Public Health.

[18] Moon J, Mun Y (2024). The association between per- and polyfluoroalkyl substances (PFASs) and brain, esophageal, melanomatous skin, prostate, and lung cancer using the 2003–2018 US National Health and Nutrition Examination Survey (NHANES) datasets. Heliyon.

[19] Smith RA, Mettlin CJ, Eyre H.

[20] Xiang F (2014). Incidence of nonmelanoma skin cancer in relation to ambient UV radiation in white populations, 1978–2012:empirical relationships. JAMA Dermatol.

[21] Stratigos A (2015). Diagnosis and treatment of invasive squamous cell carcinoma of the skin: European consensus-based interdisciplinary guideline. Eur J Cancer.

[22] Hyeraci M (2023). Systemic photoprotection in melanoma and non-melanoma skin cancer. Biomolecules.

[23] Peris K (2018). Cutaneous squamous cell carcinoma: Italian guidelines by SIDeMaST adapted to and updating EADO/EDF/EORTC guidelines. G Ital Dermatol Venereol.

[24] Centers for Disease Control and Prevention

[25] He L (2025). Arsenic exposure and risk of skin cancer (melanoma and non-melanoma): a systematic review and meta-analysis. An Bras Dermatol.

[26] International Agency for Research on Cancer.

[27] Westgard JO (1981). A multi-rule Shewhart chart for quality control in clinical chemistry. Clin Chem.

[28] Caudill SP, Schleicher RL, Pirkle JL (2008). Multi-rule quality control for the age-related eye disease study. Stat Med.

[29] AbdulMajeed J (2024). Use of conditional estimates of effect in cancer epidemiology: an application to lung cancer treatment. Cancer Epidemiol.

[30] Duan C (2021). Reflection on modern methods: understanding bias and data analytical strategies through DAG-based data simulations. Int J Epidemiol.

[31] Puhr R (2017). Firth’s logistic regression with rare events: accurate effect estimates and predictions?. Stat Med.

[32] White IR, Royston P, Wood AM (2011). Multiple imputation using chained equations: issues and guidance for practice. Stat Med.

[33] Giganti MJ, Shepherd BE (2020). Multiple-imputation variance estimation in studies with missing or misclassified inclusion criteria. Am J Epidemiol.

[34] Murad MH (2018). External validity, generalisability, applicability and directness: a brief primer. BMJ Evid Based Med.

[35] Zar LA (2025). From significance to divergence: guiding statistical interpretation through language. Curr Opin Epidemiol Public Health.

[36] StataCorp

[37] Messmer MF (2022). Risk of cancer in a community exposed to per- and poly-fluoroalkyl substances. Environ Health Insights.

[38] Li S (2025). Associations between per- and polyfluoroalkyl substances (PFAS) and county-level cancer incidence between 2016–2021 and incident cancer burden attributable to PFAS in drinking water in the United States. J Expo Sci Environ Epidemiol.

[39] Ding C (2024). PFAS promotes colorectal cancer progression via regulating RIG-I-mediated innate immune signalling. Mol Immunol.

[40] Pedersen JE (2024). Cancer incidence in a cohort of Danish firefighters: an extended long-term follow-up 1968–2021. Am J Ind Med.

[41] Yang J (2024). Perfluoroalkyl and polyfluoroalkyl substances and cancer risk: results from a dose-response meta-analysis. J Environ Health Sci Eng.

[42] Steenland K, Winquist A (2021). PFAS and cancer: a scoping review of the epidemiologic evidence. Environ Res.

[43] Baykan H, Cihan YB, Ozyurt K (2015). Roles of white blood cells and subtypes as inflammatory markers in skin cancer. Asian Pac J Cancer Prev.

[44] Jones LE (2025). Maternal exposure to legacy PFAS compounds PFOA and PFOS is associated with disrupted cytokine homeostasis in neonates: the Upstate KIDS study (2008–2010). Environ Int.

[45] Wong DA (2000). Cytokine profiles in spontaneously regressing basal cell carcinomas. Br J Dermatol.

[46] Didona D (2018). Non-melanoma skin cancer pathogenesis overview. Biomedicines.

[47] Ostrand-Rosenberg S (2008). Immune surveillance: a balance between protumor and antitumor immunity. Curr Opin Genet Dev.

[48] Leung Y-K (2018). Identification of sex-specific DNA methylation changes driven by specific chemicals in cord blood in a Faroese birth cohort. Epigenetics.

[49] García-Guede Á, Vera O, Ibáñez-de-Caceres I (2020). When oxidative stress meets epigenetics: implications in cancer development. Antioxidants.

[50] Kim S, Thapar I, Brooks BW (2021). Epigenetic changes by per- and polyfluoroalkyl substances (PFAS). Environ Pollut.

